# Identification of risk factors for elevated serum IgG4 levels in subjects in a large-scale health checkup cohort study

**DOI:** 10.3389/fimmu.2023.1124417

**Published:** 2023-03-08

**Authors:** Yoshika Tsuji, Tomohiro Koga, Fumiaki Nonaka, Kenichi Nobusue, Shin-ya Kawashiri, Hirotomo Yamanashi, Takahiro Maeda, Kazuhiko Arima, Kiyoshi Aoyagi, Meiko Takahashi, Shuji Kawaguchi, Fumihiko Matsuda, Hiroshi Fujii, Mitsuhiro Kawano, Hiroyuki Nakamura, Atsushi Kawakami, Mami Tamai

**Affiliations:** ^1^ Department of Immunology and Rheumatology, Graduate School of Biomedical Sciences, Nagasaki University, Nagasaki, Nagasaki, Japan; ^2^ Center for Bioinformatics and Molecular Medicine, Graduate School of Biomedical Sciences, Nagasaki University, Nagasaki, Nagasaki, Japan; ^3^ Department of Island and Community Medicine, Graduate School of Biomedical Sciences, Nagasaki University, Nagasaki, Nagasaki, Japan; ^4^ Department of Community Medicine, Graduate School of Biomedical Sciences, Nagasaki University, Nagasaki, Nagasaki, Japan; ^5^ Department of General Medicine, Graduate School of Biomedical Sciences, Nagasaki University, Nagasaki, Nagasaki, Japan; ^6^ Leading Medical Research Core Unit, Nagasaki University Graduate School of Biomedical Sciences, Nagasaki University, Nagasaki, Nagasaki, Japan; ^7^ Department of Public Health, Graduate School of Biomedical Sciences, Nagasaki University, Nagasaki, Nagasaki, Japan; ^8^ Center for Genomic Medicine, Graduate School of Medicine, Kyoto University, Kyoto, Kyoto, Japan; ^9^ Division of Rheumatology, Department of Internal Medicine, Kanazawa University Hospital, Kanazawa, Ishikawa, Japan; ^10^ Department of Hygiene and Public Health, Graduate School of Medical Science, Kanazawa University, Kanazawa, Ishikawa, Japan

**Keywords:** IgG4-related disease, health checkup, IgG4, magnetic bead panel assay, smoking

## Abstract

**Introduction:**

To allow the identification of IgG4-related disease (IgG4-RD) from a subclinical phase as it is important to understand the risk of elevated serum IgG4 levels. We planned to evaluate serum IgG4 levels in the participants of the Nagasaki Islands Study (NaIS), a large-scale health checkup cohort study.

**Methods:**

This study included 3,240 individuals who participated in the NaIS between 2016 and 2018 and consented to participate in the study. Serum IgG4, IgG, and IgE levels and human leukocyte antigen (HLA) genotyping results of the NaIS subjects as well as lifestyle habits and peripheral blood test results were analyzed. The magnetic bead panel assay (MBA) and the standard nephelometry immunoassay (NIA) were used to measure serum IgG4 levels. The data were evaluated using multivariate analysis to identify lifestyle and genetic factors associated with elevated serum IgG4 levels.

**Results:**

Serum IgG4 levels measured with the NIA and MBA showed a tight positive correlation between the two groups (correlation coefficient 0.942). The median age of the participants in the NaIS was 69 years [63–77]. The median serum IgG4 level was 30.2 mg/dL [IQR 12.5–59.8]. Overall, 1019 (32.1%) patients had a history of smoking. When the subjects were stratified into three groups based on the smoking intensity (pack-year), the serum IgG4 level was significantly higher among those with a higher smoking intensity. Accordingly, the multivariate analysis identified a significant relationship between smoking status and serum IgG4 elevation.

**Conclusion:**

In this study, smoking was identified as a lifestyle factor correlating positively with elevated serum IgG4 levels.

## 1 Introduction

IgG4-related disease (IgG4-RD) is a relatively new disease concept that has emerged in the 21^st^ century in Japan (1–3). IgG4-RD is an inflammatory disease that is accompanied by immune-mediated fibrosis and is characterized by elevated serum IgG4 levels and organ invasion by IgG4-positive plasma cells (4).

IgG4-RD is classified and diagnosed according to the American College of Rheumatology/European League Against Rheumatism classification criteria for IgG4-RD (5) and the 2020 Revised Comprehensive Diagnostic Criteria for IgG4-RD (6) by the Japan College of Rheumatology. The serum IgG4 level is a major determining factor for classification and diagnosis of IgG4-RD in both sets of criteria. A genome-wide association study in Japanese patients with IgG4-RD (7) has identified the human leukocyte antigen (*HLA)-DRB1* region as an IgG4-RD susceptibility locus and has shown significant associations of IgG4-RD manifestation with DRB1*04:06, DRB1*09:01, and DQB1*03:03. In these alleles, the disease risk increases (odds ratio [OR] 2.01) when the 7^th^ amino acid residue of the antigen-presenting Gβ domain of the HLA-DRB1 protein (DRB1-GB-7) is valine. In addition to HLA-DRB1*04:06, HLA-DRB1*04:03, HLA-DRB1*04:05, and HLA-DRB1*04:10 can pose disease risk when the DRB1-GB-7 amino acid residue is valine. While several clinical studies on IgG4-RD have been reported, few studies analyzing the lifestyle factors and genetic predispositions associated with serum IgG4 levels and their elevation in healthy people have been reported. No large-scale analyses or genetic predisposition analyses have been performed in this field. Small-scale studies have shown higher serum IgG4 levels in men and elderly subjects (8) A study analyzing 413 people from a public general resident database in Spain (9) has shown that IgG4 was elevated in five people (1.2%) and that higher IgG4 levels were found in men and younger subjects. However, no associations of IgG4-RD with common lifestyle habits, such as smoking, alcohol consumption, obesity, and metabolic syndrome, have been shown. In other words, lifestyle factors or genetic predispositions that induce elevated serum IgG4 levels have been predicted to exist but have not yet been identified because no large-scale analyses in healthy people have been conducted. Direct associations of good prognoses of IgG4-RD with early diagnosis and treatment initiation have been reported (10, 11). Exploring the lifestyle and genetic factors associated with elevated serum IgG4 levels is an important research subject because information useful for early diagnosis of IgG4-RD may be obtained.

Multiple methods are available for measuring IgG4 levels. The nephelometry immunoassay (NIA) is the predominantly used method, but it requires approximately 0.5 mL each of serum samples. A large-scale cohort is often used for multiple research purposes. The NaIS cohort, which was used in this study, is also a multipurpose large-scale cohort, and only a limited volume of serum sample is available for a single test. Meanwhile, the magnetic bead panel assay (MBA; Merck KGaA, Germany) requires 2 µl each of serum samples (i.e., only 1/1000 as much as the volume required for the NIA) for quantitative IgG4 measurement; however, the MBA has not been evaluated with human serum samples. Hence, in this study, we compared serum IgG4 measurements with the NIA and MBA to validate the use of the latter for estimating IgG4 in human serum samples. Based on the result, we measured serum IgG4 levels in the multipurpose, large-scale NaIS cohort and attempted to identify lifestyle and genetic factors associated with elevated serum IgG4 levels in healthy adults.

## 2 Materials and methods

### 2.1 Data collection and laboratory measurements

We conducted a community-based prospective cohort study [the Nagasaki Island Study (NaIS)] involving annual medical health checkups in Goto City, Nagasaki Prefecture, in the western part of Japan (12). This large-scale multipurpose cohort is also used for a collaborative multipurpose cohort study with Kanazawa University and Chiba University, and the analysis of serum IgG4 levels in this study was a focused research project in the collaboration. Of the 3,623 individuals who participated in the study between 2016 and 2018, 3,240 were included in the present analysis. The study protocol was approved by the institutional review boards of Kanazawa University and Nagasaki University (project registration number: 20141002-13; Nagasaki, Japan. 2016-376 (1491); Kanazawa, Japan). Written informed consent was obtained from each participant in the study, which conformed to the current Helsinki Declaration.

The MBA was used to measure the IgG4 levels in serum samples from the large-scale NaIS cohort. Prior to this assay, we compared the serum IgG4 levels measured with the NIA-based IgG subclass BS-NIA IgG4 kit (Binding Site, Birmingham, United Kingdom) and the MBA-based MILLIPLEX^®^ MAP Human Isotyping Magnetic Bead Panel kit (Merck, New Jersey, USA) using 947 samples collected from participants in Ishikawa Prefecture resident health checkups conducted by Kanazawa University (142, 582, and 223 samples from participants in 2014, 2015, and 2017, respectively). We assessed the correlation between serum IgG4 measurements with the two methods and calculated the MBA-based serum IgG4 level corresponding to the cutoff value in the NIA (the upper limit of the normal range: 135 mg/dL). Of the 3,623 samples, 383 samples was excluded due to lack of serum samples for measuring serum IgG4 levels. Subsequently, serum IgG4 levels in 3240 NaIS samples (1300, 1412, and 528 samples in 2016, 2017, and 2018, respectively) were measured with the MBA. According to the protocol, the MBA measurement was repeated at a higher sample dilution, as appropriate, when the median fluorescence value of a sample was equal to or greater than the median fluorescence value at the highest concentration used for the calibration curve. Similarly, the MBA measurement was repeated at a lower dilution, as appropriate, when the median fluorescence value of a sample was equal to or smaller than the limit of detection. An elevated serum IgG4 level was defined as an MBA-based serum IgG4 level higher than the cutoff value in the NIA (the upper limit of the normal range: 135 mg/dL). Nevertheless, 74 samples were excluded from the analysis because HLA genotyping did not yield accurate values. Finally, we utilized 3,166 samples for this project.

Among the NaIS samples, serum total IgG and IgE levels were also measured with the MBA for 200 samples in the elevated serum IgG4 group and 300 samples selected from the normal serum IgG4 group using Monte Carlo simulation with the assumption of uniform distribution. The total IgG and IgE levels were measured with the Bio-Plex Pro Human IgG Total Isotyping Assay (Bio-Rad, California, USA) and Milliplex^®^ Human Immunoglobulin IgE Single Plex Magnetic Bead Kit (Merck, New Jersey, USA), respectively, according to the measurement protocols. All assays were performed at our laboratory according to the respective manufacturer’s instructions.

HLA genotypes were analyzed by the Center for Genomic Medicine, Graduate School of Medicine, Kyoto University (7).

Nagasaki Prefecture, particularly Goto City, in which the participants of our cohort reside, is a highly endemic region for human T-cell leukemia virus type 1 (HTLV-1). There are at least 5–10 million HTLV-1-infected people in the world, including approximately 1 million infected people in Japan (13). In Goto City, Nagasaki Prefecture, the reported HTLV-1 positive rates are very high, ranging from 16.5% to 18% (12, 14), and the NaIS has also delivered many findings about HTLV-1 (15–17). HTLV-1, a human retrovirus inducing lymphoproliferative disorders, is the causative agent of adult T-cell leukemia/lymphoma and may be an environmental factor for autoimmune diseases in the NaIS cohort (18). We also analyzed the presence or absence of anti-HTLV-1 antibodies to determine the prevalence of HTLV-1 infection in this cohort study. The presence or absence of anti-HTLV-1 antibodies was evaluated with a chemiluminescent enzyme immunoassay (CLEIA) kit (Fujirebio Inc., Tokyo, Japan).

### 2.2 Lifestyle and genetic factors evaluated for association with the serum IgG4-positive status

In this study, we evaluated the lifestyle and metabolic factors according to a previously reported Spanish cohort study (9). In brief, for participants falling into the category of present or previous smokers (at least one cigarette per day), the participant-reported number of years of smoking and number of cigarettes smoked per day were used to calculate the smoking intensity (pack-years; calculated by multiplying the number of packs of cigarettes smoked per day by the number of years the person has smoked). Non-smokers, light smokers, and heavy smokers were defined as those who smoked 0, from >0 to <20, and ≥20 pack-years, respectively (19). Regarding the alcohol consumption status, the participants were asked if they were lifestyle alcohol drinkers, the mean number of times of alcohol drinking per week, and the amount of alcohol consumption per time as 500 mL beer equivalent, and the amount of alcohol consumption, g/week, was calculated. Participants undergoing treatment for hypertension and diabetes mellitus were considered to have hypertension and diabetes mellitus, respectively. Body mass index was calculated as weight (kg) ÷ (height m)^2^. Of the blood test parameters, uric acid, serum creatinine, estimated glomerular filtration rate (eGFR), and low-density lipoprotein-cholesterol values were evaluated in this study.

Based on the findings of Terao et al. (7) and Koneczny et al. (20), the HLA genotypes DRB1*03:01,*04:06, *04:03, *04:05, *04:10 and *15:01 were considered as disease susceptibility genes and DRB1*09:01 and DQB1*03:03 considered as disease prevention genes, and their associations with the serum IgG4-positive status were examined.

### 2.3 Statistical analyses

JMP^®^ Pro 16.0.0 was used for all analyses. Spearman’s rank correlation test was used to compare the measurements with the NIA and those with the MBA. The MBA-based measurement corresponding to the NIA-based cutoff value (135 mg/dL) was determined to maximize Youden’s J statistic on the receiver operating characteristic curve(ROC). The MBA-based cutoff value were used to determine the sensitivity and specificity. Wilcoxon’s rank sum test was used to determine the associations of the serum IgG4 levels with the clinical items that were continuous variables and the smoking intensity. Pearson’s chi-square test was used to determine the associations of the serum IgG4 levels with the clinical items that were binary variables. Statistical significance was defined as p < 0.05. The items for which p-values were <0.05 in the univariate analysis, except for pack-years, were used in the multivariate logistic regression analysis. The significance threshold for HLA susceptibility alleles was considered significant for all statistical testing. The significance threshold for HLA susceptibility alleles was set using the Bonferroni correction at p = 8.3 × 10^–3^, because six HLA alleles were compared.

## 3 Results

### 3.1 Correlation analysis between serum IgG4 levels measured with the NIA and the MBA

**Figure 1:**
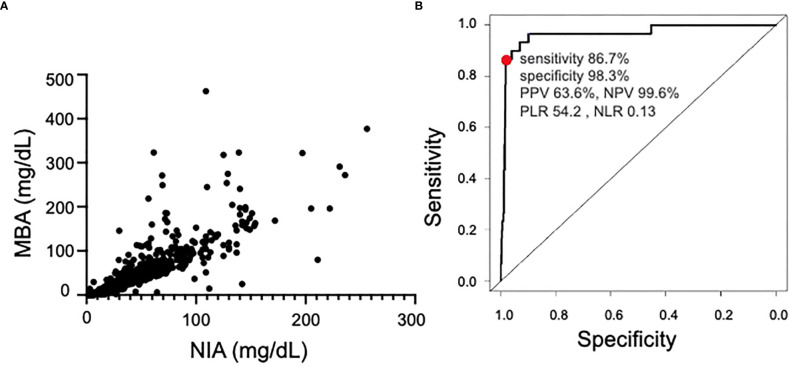
Comparison of NIA and MBA. **(A)** Correlation between NIA and MBA measurements. **(B)** Receiver operating characteristic curve of the MBA in samples with >135 mg/dL IgG4 as measured with the NIA. Area Under the Curve was 0.97. NIA, nephelometry immunoassay; MBA, magnetic bead panel assay; PPV, positive predictive value; NPV, negative predictive value; PLR, positive likelihood ratio; NLR, negative likelihood ratio.

Serum IgG4 levels in 951 serum samples from Kanazawa University were measured with the NIA and MBA. Subject demographics were as follows: 439 men (46.2%); 512 women (53.8%); and median age, 62 (interquartile range [IQR] 54–69) years. The correlation of serum IgG4 levels between the NIA group and the MBA group showed a very tight positive correlation (correlation coefficient 0.942, p < 0.0001. **Figure 1A**). The serum IgG4 levels measured with the NIA for 30 samples (3.15%) were equal to or greater than the cutoff value (135 mg/dL), which is a comprehensive criterion. Using the receiver operating characteristic curve, the MBA-based IgG4 level corresponding to the NIA cutoff value (135 mg/dL) was calculated to be 146.4 mg/dL (**Figure 1B**). IgG4 levels in 43 of the 947 samples (4.52%) were >146.3 mg/dL. Defining the true positive as an NIA-based IgG4 of >135 mg/dL, the accuracy of the MBA with IgG4 >146.3 mg/dL as the cutoff value was evaluated. The sensitivity, specificity, negative predictive value, positive predictive value, negative likelihood ratio, positive likelihood ratio and accuracy of the calculated MBA-based cutoff value were 86.7%, 98.4%, 99.6%, 63.6%, 0.13, 54.2 and 97.9%, respectively.

### 3.2 Analysis of serum IgG4 levels measured with the MBA in NaIS

A total of 3,166 subjects were enrolled in the NaIS; 1,168 were men (36.9%) and 1,998 were women (63.1%). The median serum IgG4 level was 30.4 mg/dL [IQR 12.6–59.9]. Serum IgG4 levels <146.4 mg/dL (≈NIA IgG4 <135 mg/dL) and ≥146.4 mg/dL (≈NIA IgG4 ≥135 mg/dL) were classified as the normal IgG4 group and the elevated IgG4 group, respectively. Correlations between MBA-based IgG4 levels and different items, such as lifestyle habits, patient background factors, comorbidities, and HLA alleles, were compared between the normal IgG4 group (IgG4 levels < 146.4 mg/dL) and the elevated IgG4 group (IgG4 levels ≥ 146.4 mg/dL) (**Table 1**). The number of elevated IgG4 serum samples was 200 (6.3%). The median age of the participants in the NaIS was 69 years [63–77]. Serum IgG4 levels in men and women were 37.2 mg/dL [16.8–75.6] and 26.1 mg/dL [10.7–52.8], respectively; the IgG4 level in men was significantly higher than that in women (p < 0.0001).

**Table 1:** Univariate analysis stratified by serum IgG4 level in NaIS.

	Normal IgG4 group (IgG4 levels <146.4 mg/dL)	Elevated IgG4 group (IgG4 levels ≥146.4 mg/dL)	*p* value
Number	2966	200	
Age, y.o.	69.0 (63.0–77.0)	71.0 (64.0–78.0)	0.23
Male	1058 (34.8)	110 (54.1)	<0.0001
Smoking	920 (30.3)	99 (48.8)	<0.0001
Pack-years	0.0 (0.0–8.0)	0.0 (0.0–20.0)	<0.0001
Drinking	856 (28.2)	64 (31.5)	0.33
Alcohol consumption (g/week)	0 (0.0–20.0)	0 (0.0–60.0)	0.46
Hypertension	1242 (40.9)	86 (42.4)	0.76
Diabetes Mellitus	211 (6.9)	14 (6.9)	1.0
Body mass index	22.8 (20.6–25.1)	23.0 (20.9–25.5)	0.14
Uric acid (mg/dL)	5.0 (4.2–6.0)	5.3 (4.4–6.5)	<0.01
Creatinine (mg/dL)	0.69 (0.60–0.82)	0.77 (0.63–0.88)	<0.0001
eGFR (mL/min/1.73m^2^)	70.6 (61.2–81.1)	69.4 (60.9–79.5)	0.18
LDL cholesterol (mg/dL)	119.0 (99.0–139.0)	116.0 (99.3–137.8)	0.26
HTLV-1 Ab (CLEIA)	515 (17.0)	35 (17.2)	0.95
DRB1*03:01	28 (0.94)	2 (1.0)	0.71
DRB1*04:03	133 (2.2)	6 (1.5)	0.48
DRB1*04:05	967 (15.9)	50 (12.3)	0.049
DRB1*04:06	161 (2.7)	11 (2.7)	0.87
DRB1*04:10	104 (1.7)	9 (2.2)	0.43
DRB1*09:01	846 (14.0)	57 (14.0)	1.0
DRB1*15:01	397 (13.4)	37 (18.5)	0.12
DQB1*03:03	1092 (18.0)	78 (19.2)	0.60

P-values were established using Pearson’s chi-square test or Wilcoxon’s rank sum test.

Data are median (IQR) or n (%). eGFR, estimated glomerular filtration rate; LDL cholesterol, low-density lipoprotein-cholesterol; HTLV-1, Human T-cell leukemia virus type 1. p < 0.05 was considered statistically significant.

**Figure 2:**
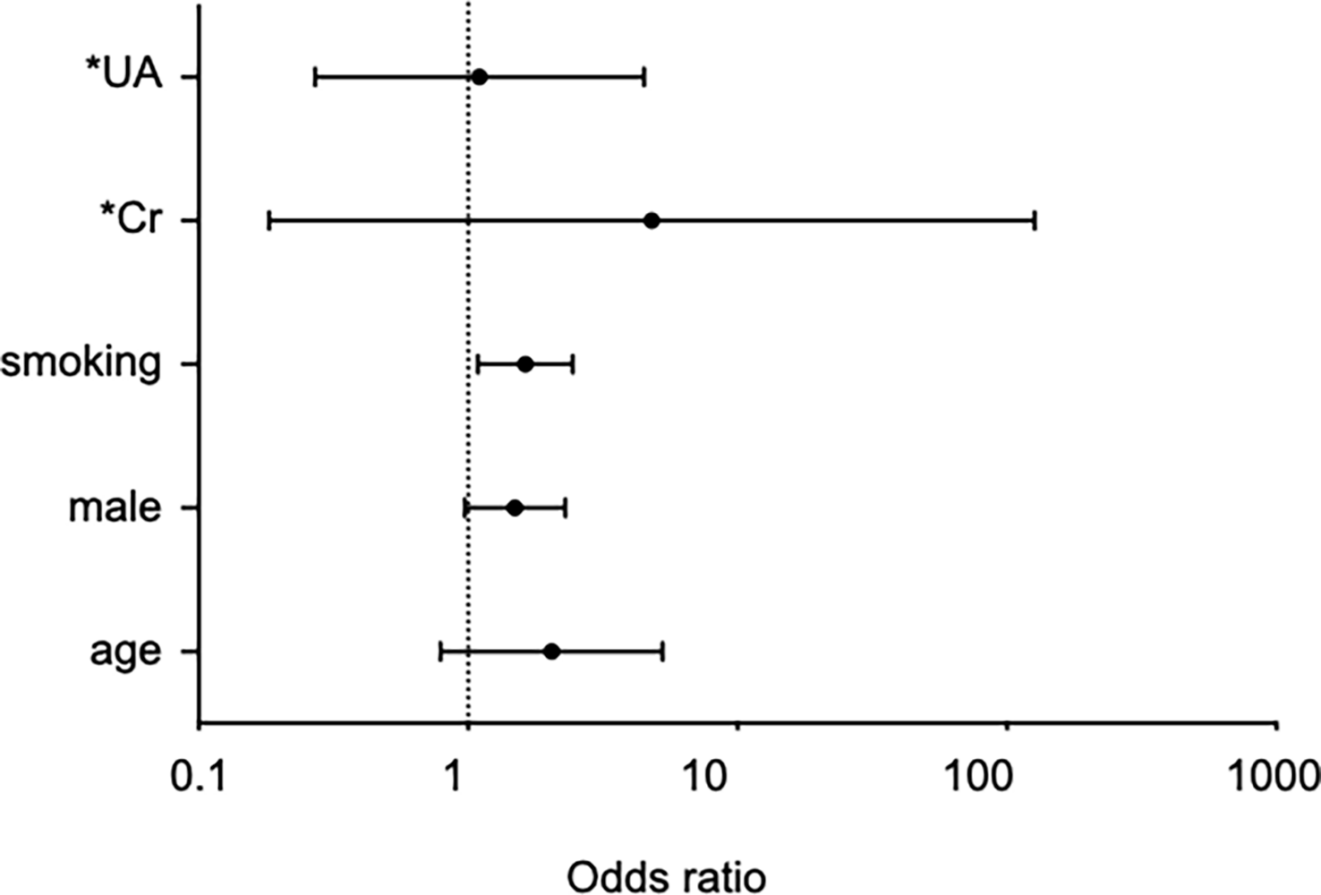
Forest plot summarizing multivariate logistic regression models for risk factor of elevated serum IgG4 levels. *Odds ratios for continuous variables are values that have changed over the entire range. UA, uric acid, Cr, creatinine. The x-axis shows odds ratios and the error bars show 95% confidence intervals.

**Figure 3:**
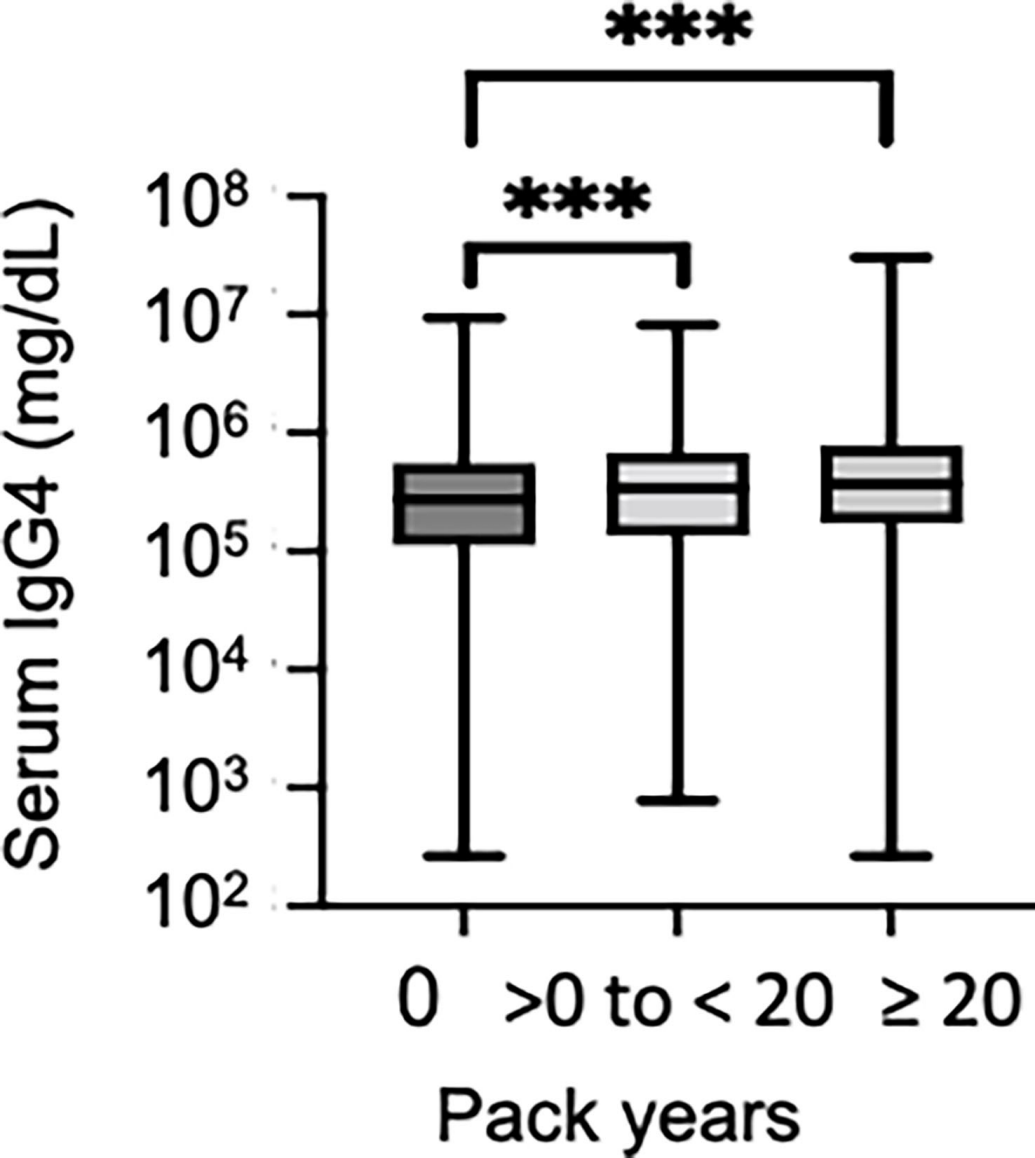
Comparison of serum IgG4 levels by smoking intensity. Serum IgG4 levels divided into the following three categories according to the smoking intensity were compared: pack-year = 0 non-smoker, 0 < pack-year < 20 light smoker, pack-year ≥ 20 heavy smoker. ***p < 0.001 with respect to the reference category.

The univariate analysis of IgG4 levels revealed that the elevated IgG4 group was characterized by statistically significantly greater proportions accounted for by men and smokers (p < 0.001) and statistically significantly higher smoking intensity, uric acid level, and serum creatinine level. The elevated serum IgG4 levels were not associated with anti-HTLV-1 antibodies, previously reported IgG4-RD susceptibility genes (HLA-DRB1*03:01,*04:06, *04:03, *04:05, *04:10, *15:01), or resistance genes (HLA-DRB1*09:01, DQB1*03:03). The multivariate analysis conducted with clinical items showing significant differences in the univariate analysis, except for the smoking intensity (pack-years), revealed a significant association between the smoking status and serum IgG4 elevation OR 1.63, 95% confidence interval [CI] 1.09–2.45, p < 0.05, **Figure 2**). Serum IgG4 levels were compared among the three smoking-intensity-based groups: non-smoker (n = 2151), light smoker (n = 513), and heavy smoker (n = 459) groups. Serum IgG4 levels in the non-smoker, light smoker, and heavy smoker groups were 27.4 mg/dL [IQR 11.2–55.1], 33.9 mg/dL [IQR 13.5–68.4] and 37.7 mg/dL [17.9–80.4] (**Figure 3**), respectively. The serum IgG4 levels of the light smoker group and the heavy smoker group were significantly higher than that of the non-smoker group (p = 0.0003 and <0.0001, respectively), and the serum IgG4 level of the heavy smoker group tended to be higher than that of the light smoker group (p = 0.0501). Therefore, the serum IgG4 level tended to increase with the smoking intensity (**Figure 3**). The serum total IgG levels, IgG4/total IgG ratios, and IgE levels were compared between 200 elevated IgG4 serum samples and 300 serum samples randomly selected from the normal IgG4 serum samples (**Table 2**). The total IgG level of the elevated serum IgG4 group was significantly higher, and so were the IgG4/total IgG ratio and IgE (p = 0.020, <0.0001). In the 200 samples in the elevated serum IgG4 group and the 300 randomly selected samples in the normal serum IgG4 group, the total IgG level did not differ significantly between smokers and non-smokers (p = 0.31); however, the IgG4/total IgG ratio (0.13 vs. 0.32, p < 0.0001) and serum IgE level (553.7 vs. 826.1, p < 0.0001) of the smokers were significantly higher (**Table 3**).

**Table 2:** Univariate analysis stratified by serum IgG4 level and IgG subclass in NaIS.

	Normal IgG4 group (IgG4 levels <146.4 mg/dL)	Elevated IgG4 group (IgG4 levels ≥146.4 mg/dL)	*p* value
Number	300	200	
Age, y.o.	70.0 (63.0–77.0)	71.0 (64.0–78.0)	0.22
Male	114 (38.0)	110 (55.0)	<0.01
Smoking	98 (32.7)	99 (49.5)	<0.01
Pack-year	0.0 (0.0–11.1)	0.0 (0.0–20.0)	<0.01
Drinking	89 (29.7)	64 (32.0)	0.40
Alcohol consumption (g/week)	0 (0.0–40.0)	0 (0.0–60.0)	0.74
Hypertension	110 (36.7)	56 (28.0)	0.052
Diabetes Mellitus	20 (6.7)	10 (5.0)	0.56
Body mass index	22.8 (20.5–24.9)	23.0 (20.9–25.5)	0.063
Uric acid (mg/dL)	5.0 (4.2–6.0)	5.3 (4.4–6.5)	0.0073
Creatinine (mg/dL)	v0.69 (0.60–0.84)	0.77 (0.63–0.88)	0.010
eGFR (mL/min/1.73m^2^)	70.7 (60.9–81.5)	69.4 (60.1–79.5)	0.32
LDL cholesterol (mg/dL)	118.0 (100.2–138.0)	116.0 (99.3–137.8)	0.55
HTLV-1 Ab (CLEIA)	53 (17.9)	35 (17.5)	1.0
Total IgG (mg/dL)	375.3 (308.2–443.3)	394.1 (335.0–469.9)	0.020
IgG4/total IgG	0.066 (0.029–0.14)	0.55 (0.44–0.81)	<0.0001
IgE (ng/mL)	556.9 (293.2–1031.7)	892.7 (455.1–1656.2)	<0.001

P-values were established using Pearson’s chi-square test or Wilcoxon’s rank sum test.

Data are median (IQR) or n (%). eGFR, estimated glomerular filtration rate; LDL cholesterol, low-density lipoprotein-cholesterol; HTLV-1, Human T-cell leukemia virus type 1. p < 0.05 was considered statistically significant.

**Table 3:** Univariate analysis stratified by smoking in NaIS.

	Non-smoker	Smoker	*p* value
Number	303	197	
Age, y.o.	72.0 (65.0–78.0)	69.0 (61.0–76.5)	0.0045
Male	52 (17.1)	172 (87.3)	<0.0001
Drinking	51 (16.8)	102 (51.8)	<0.0001
Alcohol consumption	0 (0.0–0.0)	20.0 (0.0–140.0)	<0.0001
Hypertension	99 (32.7)	67 (34.0)	0.77
Diabetes Mellitus	16 (5.3)	14 (7.1)	0.34
Body mass index	22.6 (20.2–25.2)	23.3 (21.2–25.3)	0.048
Uric acid (mg/dL)	4.8 (4.0–5.6)	5.7 (4.7–6.7)	<0.0001
Creatinine (mg/dL)	0.66 (0.58–0.78)	0.83 (0.73–0.94)	<0.0001
eGFR (mL/min/1.73m2)	70.4 (61.4–81.0)	70.0 (60.0–80.3)	0.93
LDL cholesterol (mg/dL)	121.0 (103.0–141.0)	113.0 (96.5–130.5)	0.0023
HTLV-1 Ab (CLEIA)	61 (20.1)	27 (13.7)	0.09
Total IgG (mg/dL)	3826 (3123.4–4455.1)	3873.5 (3214.2–4659.5)	0.31
IgG4/total IgG	0.13 (0.039–0.46)	0.32 (0.078–0.54)	<0.0001
IgE (ng/mL)	553.7 (307.8–1020.1)	826.1 (467.1–1867.2)	<0.0001

P-values were established using Pearson’s chi-square test or Wilcoxon’s rank sum test.

Data are median (IQR) or n (%). eGFR, estimated glomerular filtration rate; LDL cholesterol, low-density lipoprotein-cholesterol; HTLV-1, Human T-cell leukemia virus type 1. p < 0.05 was considered statistically significant.

## 4 Discussion

IgG4-RD can cause fibroinflammatory lesions in almost any organ. The survival rate for IgG4-RD are good because treatment with glucocorticoids is generally successful; however, diminished treatment responses have been reported in patients with a long disease duration and in those with IgG4-related kidney disease with advanced fibrosis at the time of diagnosis (10, 11). Thus, identification of subclinical IgG4-RD in health checkups is crucial so that therapeutic intervention may be initiated early. In the American College of Rheumatology/European League Against Rheumatism classification criteria for IgG4-RD (5) and the 2020 Revised Comprehensive Diagnostic Criteria for IgG4-RD (6) by the Japan College of Rheumatology, IgG4-RD is classified or diagnosed comprehensively based on clinical findings, imaging findings, serum IgG4 levels, and histological findings. Nevertheless, in this study, we focused on factors associated with elevated serum IgG4 levels because we aimed to identify the population at high risk for IgG4-RD from among people undergoing health checkups. We used the MBA to measure serum IgG4 levels because it requires only a small amount of serum and is suitable for analysis in large-scale multipurpose cohorts.

This study was the first to test the correlation between serum IgG4 levels measured with the MBA and those measured with the NIA, which is the primary serum IgG4 assay method employed in clinical settings. The comparison of serum IgG4 levels measured with the MBA and the NIA demonstrated a very tight correlation between the two sets of values. The comparison also showed that the IgG4 cutoff values with the MBA and the NIA were 146.4 mg/dL and 135 mg/dL, respectively, and that the accuracy was very high. As only 2 µL of serum was required to measure the IgG4 level with the MBA, this method appeared to be suitable for serum IgG4 measurement using a small sample volume, thus providing a meaningful advantage. Serum total IgG levels are known to interfere with serum IgG4 levels. The serum IgG4/total IgG ratio of the elevated serum IgG4 group was significantly higher than that of the normal serum IgG4 group, thereby suggesting the usefulness of IgG4 measurement with the MBA.

Only one previous study in Spain has reported the analysis of serum IgG4 levels in 413 general residents in a public database (9). The median serum IgG4 level reported therein was 33.9 mg/dL, which agreed with the value in our report. However, the proportion of the subjects with serum IgG4 levels higher than the cutoff value (135 mg/dL) was 1.2%, which was lower than the percentage in our report. This difference may be attributed to subject demographics, such as race and age (median age, 70 years in our study and 59 years in the Spanish study), differing between the two studies.

Like the Spanish study, our study also analyzed the associations between serum IgG4 levels and lifestyle factors. In the Spanish study, the serum IgG4 level of smokers was higher than that of non-smokers in the univariate analysis but not in the multivariate analysis with adjustments. In contrast, our multivariate analysis revealed positive correlations between smoking and elevated serum IgG4 as well as between smoking intensity and serum IgG4 levels. This difference may be due to a large variation in the number of samples analyzed (413 vs. 3,166). A previous study has reported that patients with IgG4-RD were 1.8 times more likely to be smokers than those without IgG4-RD [OR 1.79 (95% CI 1.08–2.95), p = 0.02] (21). Moreover, cigarette smoking is known to activate Th2 cytokine cascades more strongly than the Th1 response (22). Airway epithelial cells are the main source of thymic stromal lymphopoietin (TSLP), IL-25, and IL-33 (23), which govern upstream of the canonical Th2 cytokines. The production of serum IgG4 is stimulated by Th2 cytokines (24). These data are consistent with our present correlation of smoking with serum IgG4 levels, and we speculate that smoking is a risk factor for IgG4 elevation, thus leading to the development of IgG4-RD.

In the Spanish study (9), there were associations between serum IgG4 levels and allergy traits and serum IgG4 and IgE levels were significantly correlated. The participants in our cohort were not requested to report previous allergies and did not undergo skin prick test or aeroallergen-specific IgE measurement. Nonetheless, the serum IgE level of the elevated serum IgG4 group was also significantly higher than that of the normal serum IgG4 group in our cohort. Given the higher frequency of history of allergy and elevated serum IgE level in patients with IgG4-RD, this result suggests that the elevated serum IgG4 group in our cohort included individuals with subclinical IgG4-RD.

Previous studies searching for Ig4-RD associated genetic factors from among limited candidate genes have reported *HLA* (25), *FCRL3* (26), *CTLA4* (27), and *KCNA3* (28) to be the disease-associated genes. A genome-wide comprehensive analysis of 857 cases in a multicenter study by 50 Japanese research/medical institutions to which IgG4-RD specialists belonged showed significant correlations of IgG4-RD with *HLA-DRB1* and *FRGR2B* genes, with the OR of the former being particularly higher than that of the latter (7). In the present study, we analyzed the disease susceptibility genes (HLA-DRB1*03:01,*04:06, *04:03, *04:05, *04:10, *15:01) and resistance genes (HLA-DRB1*09:01, DQB1*03:03) identified in the genome-wide comprehensive analysis but found no associations with serum IgG4 elevation (7, 20). This observation is likely because the genetic background of IgG4-RD was not concentrated in the subjects of this study who were healthy people.

The NaIS cohort analyzed in this study is a large-scale multipurpose cohort in a region with a population of approximately 35,000. While the 3,166 samples included in the present study covered approximately 9% of the regional population, the subjects had a median age of 70 years and were skewed toward an elderly population, which is a possible limitation of this study. Another limitation is that the genetic factor evaluation included only the *HLA* gene and did not include non-HLA genes, such as the *FRGR2B* gene. Nevertheless, smoking was identified as a lifestyle factor associated positively with IgG4-positive serum samples. In future, we intend to prospectively follow up subjects with serum IgG4-positivity at Goto Chuoh Hospital, which is a follow-up institution in the NaIS, and demonstrate the association between smoking and IgG4-RD. The aim is to establish an algorithm to identify subclinical IgG4-RD in health checkups.

To summarize the results of the present analysis, the MBA provided IgG4 measurements correlating tightly with IgG4 levels obtained with the NIA. The MBA required only a small volume of serum sample to measure IgG4, and the multivariate analysis identified smoking as a lifestyle factor associated significantly with elevated serum IgG4 levels. However, the HLA alleles previously reported to be associated with IgG4-RD were not identified as alleles associated significantly with elevated serum IgG4 levels in healthy people.

## Data availability statement

The data sets used and/or analyzed during the present study are available from the corresponding author on reasonable request.

## Ethics statement

The study protocol was approved by the institutional review boards of Kanazawa University and Nagasaki University (project registration number: 20141002-13; Nagasaki, Japan. 2016-376 (1491); Kanazawa, Japan). The patients/participants provided their written informed consent to participate in this study.

## Author contributions

YT involved in data analysis, interpretation of data, drafting. MaT involved in conception, data acquisition, data analysis, interpretation of data, revising. TK involved in data analysis, interpretation of data, revising. AK involved in conception, interpretation of data, revising. FN, KN, S-YK, HF, MK, HN, HY, KaA, KiA and TM involved in data acquisition, interpretation of data. MeT, SK and FM involved in data acquisition, data analysis, interpretation of data. All authors contributed to the article and approved the submitted version.
